# Recognizing Diogenes syndrome: a case report

**DOI:** 10.1186/1756-0500-7-276

**Published:** 2014-05-02

**Authors:** Jeffrey DC Irvine, Kingsley Nwachukwu

**Affiliations:** 1 Department of Academic Family Medicine, College of Medicine, University of Saskatchewan, La Ronge, Saskatchewan, Canada; 2 Department of Psychiatry, College of Medicine, University of Saskatchewan, North Battleford, Saskatchewan, Canada

**Keywords:** Diogenes syndrome, Self-neglect, Hording, Senile squalor, Mental health

## Abstract

**Background:**

Diogenes syndrome is a behavioural disorder characterized by domestic squalor, extreme self-neglect, hoarding, and lack of shame regarding one’s living condition. Patients may present due to a range of reasons. Recognizing these will allow for earlier management of this high-mortality condition.

**Case presentation:**

61-year Caucasian female known with bipolar 1 disorder presented with manic symptoms. She was very unkempt and foul smelling. After being admitted involuntarily, she requested that someone go to her home to feed her pets. Her house was filled with garbage, rotting food, and animal feces. She had no insight into any personal hygiene or public health problems.

**Conclusions:**

Patients with Diogenes syndrome may be difficult to identify. Knowledge of the characteristics of Diogenes syndrome can aid in earlier recognition of such individuals, in order to decrease morbidity and mortality, and to improve public health.

## Background

Diogenes syndrome (DS) is a behavioural disorder characterized by domestic filth, or squalor, extreme self-neglect, hoarding, and lack of shame regarding one’s living condition [1]. The approximate annual incidence of Diogenes is 0.05% in people over the age of 60 [2]. Affected individuals come from any socioeconomic status, but are usually of average or above-average intelligence [3]. It is often associated with other mental illnesses, such as schizophrenia, mania, and frontotemporal dementia [4]. While no clear etiology exists, it is hypothesized that it may be due to a stress reaction in people with certain pre-morbid personality traits, such as being aloof, or certain personality disorders, such as schizotypal or obsessive compulsive personality disorder [5,6]. There are suggestions that an orbitofrontal brain lesion may lead to such behaviours [7], while others state that chronic mania symptoms, such as poor insight, can lead to such a condition [4].

Although DS is not uniquely recognized in the Diagnostic and Statistical Manual (DSM) of Mental Disorders, the fifth version of the manual now identifies hoarding (syllogomania), as a psychiatric diagnosis [8]. Syllogomania is differentiated from Diogenes in that other characteristics such as the squalor and neglect are present in DS [9]. Mortality is increased in these patients, with a 46% five-year death rate [1], which is commonly due to physical illnesses such as pneumonia. This is subsequent to self-neglect, poor infection control practices, nutritional deficiency, and lack of presentation to medical care. These individuals self-isolate, and therefore may not be found until much later post-mortem. This can make specific causes of death difficult to determine [10]. This paper identifies that these patients can present due to a variety of reasons, and sometimes only by chance. Keeping this constellation of symptoms in mind will allow for a more prompt diagnosis and initiation of management of these clients.

## Case presentation

A 61-year-old obese Caucasian female with a previous history of bipolar 1 disorder and hypothyroidism, presented for an out-patient psychiatric follow-up review accompanied by her Community Psychiatry Nurse. She was found to have pressured speech, elated mood, increased energy, and very poor personal hygiene. She was disheveled, unkempt, wearing dirty clothes, and was foul smelling. She was very agitated, and was verbally and physically abusive to staff. She had no insight, and refused any form of treatment. She was diagnosed with having a manic relapse secondary to non-adherence to medication, and was involuntarily admitted to the in-patient psychiatric ward. Complete blood count, electrolytes, glucose, liver function, and lipid profile were all within normal limits. Thyroid stimulating hormone was slightly elevated, although T4 was within normal limits. Vitamin B12 was on the low end of normal. She was re-started on her previous psychiatric medication, namely divalproex and clonazepam.

**Figure 1:**
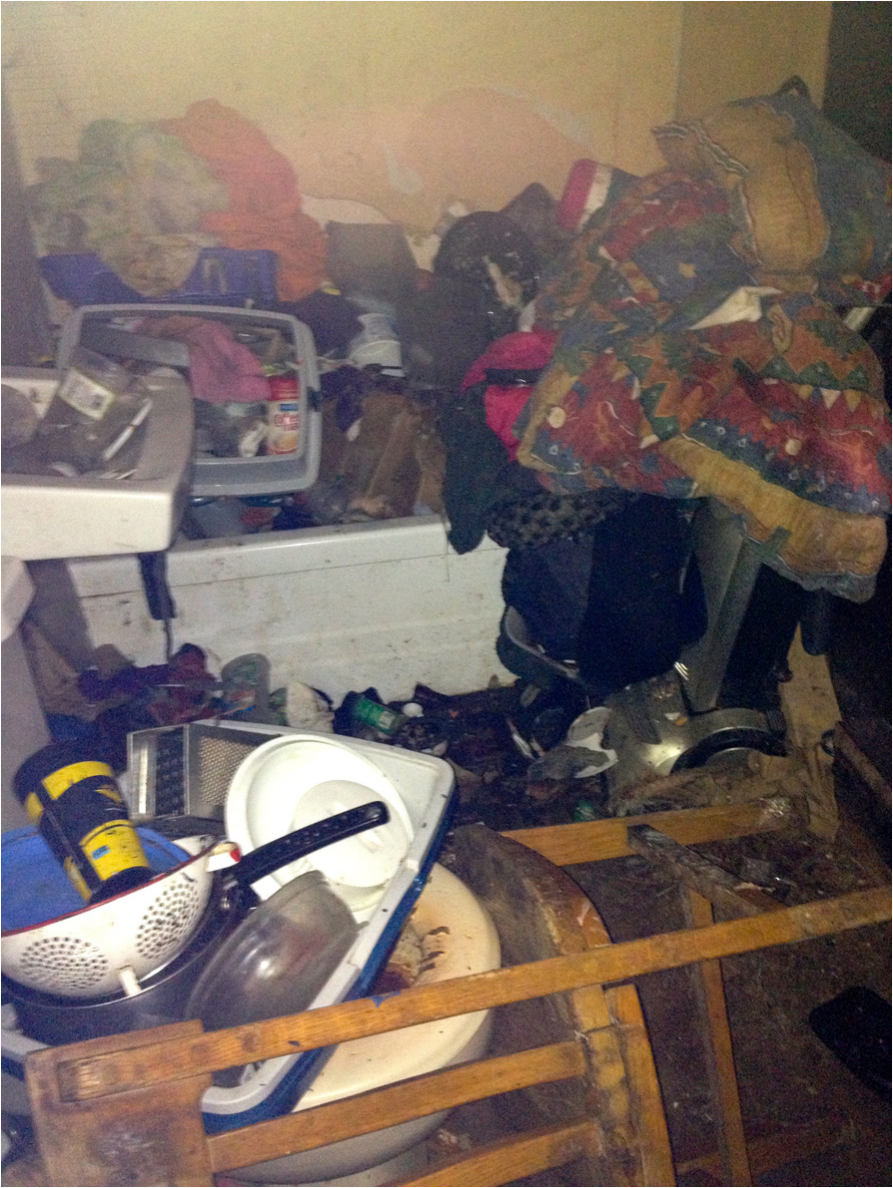
**Bathroom.** This is the state in which the bathroom was found in the patient’s house. The patient had been using the toilet for both toileting and periodically washing her dishes.

**Figure 2:**
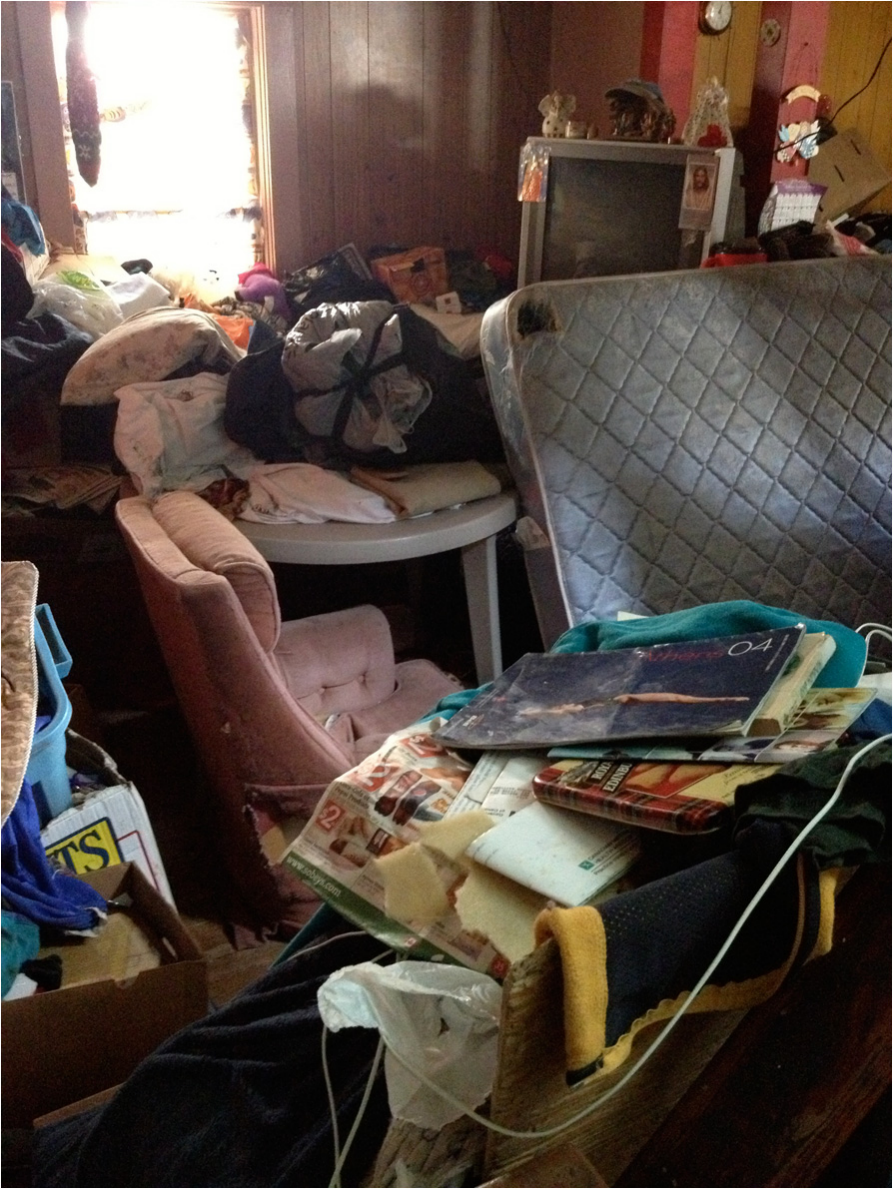
**Living room.** The patient’s living room was filled with dirty clothing, old newspaper, and animal feces.

The following day she was adamant about having to go feed her cats and dogs, and eventually gave permission for a Community Mental Health Nurse enter her house to attend to her pets. Upon entering the house, it was found to be in complete disarray. The house was crammed with filthy clothes, garbage, dirty dishes, and rotting food. There was no kitchen sink in sight, and it looked as if some dishes were being cleaned in the toilet (see Figures 1 and 2). Any clear space of floor was strewn with cat and dog feces. An unbearable stench emanated from the entire two-story home. Upon questioning the patient regarding the state of her home and personal hygiene, the patient had no insight into any problems. At this time, a diagnosis of Diogenes syndrome was suspected.

Diagnosis of DS can be difficult as no one constellation of symptoms has been established. Hoarding, which can occur in DS, can also be found in many psychiatric conditions such as obsessive-compulsive disorder (OCD), schizophrenia, dementia, and others [11]. The act of accumulation in DS is more likely ego-syntonic however, in contrast to the anxiety and intrusive thoughts that accompany collection in OCD [12]. DS can be distinguished from personality disorders in that the personality in DS deteriorates, while the true personality disorder does not [13]. Self-neglect can also be a part of dementia, schizophrenia, OCD, and affective disorders [11]. Frontal lobe dementia tends to occur approximately 10 years prior to the typical age that DS patients are affected though [13]. A diagnosis of schizophrenia can include delusions, hallucinations, and disorganized speech [14], which are not classical characteristics of DS. Clearer delineations between disorders need to exist however. An alterative suggestion was that DS “may be a final common pathway of different psychiatric disorders” [15].

Management of DS can be difficult, as patients often deny that there is a problem, may refuse any help, and can present late to medical attention [16], often in crisis. Ethical and legal issues can then arise, such as finding a balance between autonomy and beneficence [17]. For example, a patient’s notion of self-neglect can be quite different than the view of their healthcare provider [13]. Public Health issues may also arise concerning the patient’s housing. Fire, mould, and biological material can pollute the surrounding environment, so the health of nearby residents needs to be considered.

Establishing good rapport is vital in order to decrease the patient’s resistance to aid. A physical exam should be completed. Blood tests may include potassium, calcium, vitamin B12, iron, thyroid stimulating hormone, folate, and albumin [11]. Functional inquiries and cognitive testing may be useful. Treatment usually begins by looking at any other possible psychiatric issues such as mania or psychosis. Risperidone has been suggested for use in DS even when there are no underlying psychotic features [11]. Other pharmaceuticals that may be of benefit include zolpidem for sleep, paroxetine for hoarding, and sodium valproate or quetiapine for secondary bipolar disorders [11]. Flexible outpatient treatment through community care providers is preferable if there is little risk to the patient or to others [2]. This can include counseling and cleaning services, and individualized case management [18]. The mental health act can be used if difficulties are experienced in managing higher-risk patients. If management is not conducted in a sensitive manner, patients will simply return to the same living condition, with much more resistance to support and follow-up.

The prognosis of affected individuals depends on their capability of re-integrating into society, and often relies on the patients making small changes away from unhealthy living conditions [19]. Other poor prognostic factors include poor physical health, which may already be advanced due to neglect, and early age at onset.

As time progressed as an in-patient, the patient’s mood settled, although she remained guarded, with little insight into her self-care. The patient required persistent and gentle pressure in order to even start thinking about de-cluttering and improving her personal hygiene. She was eventually persuaded to allow a company to help her clean her home, at a cost of $8,073. The patient was present at the clean up. The sink was eventually found under a large pile of debris. The patient is now living at home, and receiving close follow-up with her Community Psychiatry Nurse and Psychiatrist. It remains to be seen whether these interventions will make any long-term impact to her living condition and health.

## Conclusions

This case illustrates the importance of suspecting Diogenes syndrome when elderly patients present with certain non-specific symptoms that may otherwise be disregarded. These include patients who are unkempt and malodorous, with personality traits of being unfriendly, detached, or suspicious. Such characteristics can also be related to multiple other psychiatric conditions. Timely diagnosis and respectful management may reduce both acute and chronic physical illness, increase personal and home hygiene and safety, and improve public health outcomes.

## Consent

Written informed consent was obtained from the patient for publication of this Case Report and any accompanying images. A copy of the written consent is available for review by the Editor-in-Chief of this journal.

## Competing interests

The authors have nothing to declare.

## Authors’ contributions

JDCI – Contributed to design, carried out acquisition of data, drafted the manuscript, and gave final approval of the version to be published. KN – Contributed to conception and design, revised the manuscript critically for important intellectual content, and gave final approval of the version to be published. All authors read and approved the final manuscript.

## Authors’ information

JDCI is in the second year of a rural family medicine residency training program at the University of Saskatchewan, Canada.

KN is a consultant psychiatrist in North Battleford, Saskatchewan, Canada, and faculty at the University of Saskatchewan, Canada.

## References

[B1] Badr A Hossain A Iqbal J Diogenes syndrome: when self-neglect is nearly life threatening Clin Geriatr Aug 2005 13 8 10 13

[B2] Cipriani G Lucetti C Vedovello M Nuti A Diogenes syndrome in patients suffering from dementia Dialogues Clin Neurosci 2012 14 4 455 460 23393422 10.31887/DCNS.2012.14.4/gcipriani PMC3553571

[B3] Reyes-Ortiz C Diogenes syndrome: the self-neglect elderly Comp Ther 2001 27 2 117 121 10.1007/s12019-996-0005-6 11430258

[B4] Fond G Jollant F Abbar M The need to consider mood disorders, and especially chronic mania, in cases of diogenes syndrome (squalor syndrome) Int Psychogeriatr 2011 23 3 505 507 10.1017/S1041610210001663 20836916

[B5] Rosenthal M Stelian J Wagner J Berkman P Diogenes syndrome and hoarding in the elderly: case reports Isr J Psyciatry Relat Sci 1999 36 1 29 34 10389361

[B6] Biswas P Ganguly A Bala S Nag F Choudhary N Sen S Diogenes syndrome: a case report Case Rep Dermat Med 2013 2013 595192 10.1155/2013/595192 PMC3572640 23424686

[B7] Funayama M Mimura M Koshibe Y Kato Y Squalor syndrome after focal orbitofrontal damage Cog Behav Neurol Jun 2010 23 2 135 139 10.1097/WNN.0b013e3181d746ba 20535064

[B8] American Psychiatric Association Highlights of changes from DSM-IV-TR to DSM-5 http://www.dsm5.org/Documents/changes%20from%20dsm-iv-tr%20to%20dsm-5.pdf

[B9] Zuliani G Soavi C Dainese A Milani P Gatti M **Diogenes syndrome or isolated syllogomania**? **Four heterogeneous clinical cases** Aging Clin Exp Resin press 10.1007/s40520-013-0067-0 23846849

[B10] Byard R Diogenes or havisham syndrome and the mortuary Forensic Sci Med Patholin press 10.1007/s12024-013-9458-y 23702654

[B11] Amanullah S Oomman S Datta SS “Diogenes syndrome” revisited Ger J Psychiat 2009 12 38 44

[B12] Pertusa A Frost RO Fullana MA Samuels J Steketee G Tolin D Saxena S Leckman JF Mataix-Cols D Refining the diagnostic boudaries of compulsive hoarding: a critical review Clin Psychol Rev 2010 30 371 386 10.1016/j.cpr.2010.01.007 20189280

[B13] O’abrien J Self-neglect in old age Aging Health 2011 7 4 573 581 10.2217/ahe.11.47

[B14] Amerian Psychiatric Association Schizophrenia http://www.dsm5.org/Documents/Schizophrenia%20Fact%20Sheet.pdf

[B15] Fontenelle L Diogenes syndrome in a patient with obsessive-compulsive disorder without hoarding Gen Hosp Psychiat 2008 30 288 290 10.1016/j.genhosppsych.2007.10.001 18433664

[B16] Bonci G A case of diogenes syndrome: clinical and ethical challenges JAGS Sept 2005 60 9 1780 1781 10.1111/j.1532-5415.2012.04128.x 22985153

[B17] Freckelton I Hoarding disorder and the law J Law Med Dec 2012 20 2 225 249 23431842

[B18] Snowdon J Halliday G How and when to intervene in cases of severe domestic squalor Int Psychogeriatr 2009 21 6 996 1002 10.1017/S1041610209990597 19589194

[B19] Cooney C Hamid W Review: diogenes syndrome Age Ageing 1995 24 451 453 10.1093/ageing/24.5.451 8669353

